# Improved methodology for fixation and preparation of *Aedes aegypti* embryos

**DOI:** 10.1371/journal.pone.0304802

**Published:** 2024-05-31

**Authors:** William Reid, Geyenna Sterling-Lentsch, Marc S. Halfon

**Affiliations:** 1 Department of Biochemistry, University at Buffalo-State University of New York, Buffalo, NY, United States of America; 2 Department of Biomedical Informatics, University at Buffalo-State University of New York, Buffalo, NY, United States of America; 3 Department of Biological Sciences, University at Buffalo-State University of New York, Buffalo, NY, United States of America; University of Bari: Universita degli Studi di Bari Aldo Moro, ITALY

## Abstract

The yellow fever mosquito *Aedes aegypti* is a major disease vector and an increasingly popular emerging model research organism. We present here an improved protocol for the collection, fixation, and preparation of *A. aegypti* embryos for immunohistochemical and *in situ* hybridization studies. The processing of *A. aegypti* embryos for such studies is complicated by the inability to easily remove the vitelline membrane, which prevents the reagents needed for staining from reaching their targets, and which furthermore obscures visualization of the embryo since the membrane is highly sclerotized. Previously described protocols for removal of the vitelline membrane are very low throughput, limiting the capacity of work that can be accomplished in a reasonable timeframe. Our adapted protocol increases the throughput capacity of embryos by an individual user, with experienced users able to prepare an average of 100–150 embryos per hour. The protocol provides high-quality intact embryos that can be used for morphological, immunohistochemical, and *in situ* hybridization studies. The protocol has been successfully tested on embryos of ages ranging from 14h after egg laying (AEL) at 27°C through to 55h AEL. Critical to the success of the optimized protocol is the selection, fabrication, and description of the tools required. To this end, a video-demonstrated protocol has been placed at *protocols.io* to clarify the protocol and provide easy access and training to anyone interested in the preparation of *A. aegypti* embryos for biological studies.

## Introduction

The yellow fever mosquito, *Aedes aegypti*, is a major vector for arboviruses including dengue, Zika, and chikungunya [1]. Much research has gone into developing methods of control, which could be aided by a more detailed understanding of mosquito development [2]. Studies on the development of insect embryos requires the fixation and preparation of embryos free of the vitelline membrane so that reagents such as antibodies and nucleic acid probes can gain access to their targets. The processing of the embryos of *Drosophila melanogaster* is quick and convenient as the vitelline membrane can be removed chemically. However, *A. aegypti* possesses a robust and pigmented vitelline membrane that requires mechanical peeling for each individual embryo. Although previous methods for the mechanical removal of the vitelline membrane have been published, those protocols are less amenable to higher throughput embryo processing than that presented here [3–7]. Other protocols have degraded the vitelline membrane in sodium hypochlorite; however this resulted in less than 5% of embryos being suitable for staining [8].

While the collection and fixation of *A. aegypti* embryos employs a simple methodology similar to that used for other insect species, the common bottleneck step in previously published methods is the removal of the vitelline membrane. As such, a procedure for the reliable, reproducible, and efficient removal of this membrane is needed. In our protocol, we describe improvements on the methods of vitelline membrane removal (“peeling”) to expedite embryo preparation. These improvements include using an easily-assembled jig for precise alignment of embryos, description of new tools for optimal removal of the anterior ends of the vitelline membrane, and a higher-throughput “assembly-line” approach to vitelline membrane removal. In addition, we found that whereas previously established methodologies only described single methods for embryo peeling, multiple methods of embryo peeling are actually needed to account for the dynamic nature of both the vitelline membrane and the serosal cuticle, whose physical properties change over developmental time. We therefore introduce protocol variants for embryos of different ages; one for younger embryos (<20h after egg laying (AEL) at 27°C), and one for older embryos (>20h AEL at 27°C). (Blastoderm-stage embryos have not yet been tested with our protocol, and it remains to be determined whether or not our approach improves upon the one developed by Juhn and James [4] for that developmental stage.) Finally, since the removal of the vitelline membrane, which is both difficult and tedious, is best demonstrated visually, we provide videos of every step necessary for the preparation of *A. aegypti* embryos for immunohistochemical and *in situ* hybridization studies. This includes the collection and fixation of *A. aegypti* embryos, the development-specific procedures for removal of the vitelline membrane, and the fabrication of protocol-specific tools and supplies.

In our hands, we find that approximately 100–150 older embryos, enough for a simple staining experiment, can be peeled in an hour, while the same number of younger embryos may take up to 5–6 h for the same numbers of embryos due to the differences in the methodology. These results represent a nearly ten-fold improvement over our experience using previously published methodologies.

## Materials and methods

The protocol described in this peer-reviewed article is published on *protocols.io* (doi.org/10.17504/protocols.io.261ged88wv47/v1) and is included for printing as S1 File with this article.

### Expected results

The removal of the vitelline membrane is a time-intensive step for the preparation of *A. aegypti* embryos. Confounding this issue is the dynamic nature of the vitelline membrane and the serosal cuticle, whose physical properties vary with developmental stage. First, the vitelline membranes of younger (≤20h AEL) are more fragile and prone to tearing. For these embryos, it is easier to manually remove the vitelline membrane from the embryo by creating a slit in the membrane along the sagittal plane and then peeling the membrane away from the embryo. When the embryos are older, however, the vitelline membrane is less fragile, and the embryos can be extruded from the vitelline membrane using a blunt probe. Further, since the vitelline membrane is stronger in older embryos, if they are slit along the sagittal plane, the vitelline membrane will curve inward and cut into the embryo itself. As such, it is ideal to have two approaches to peeling: one for younger, and one for older embryos.

Second, the serosal cuticle provides an additional problem for embryo peeling, since this cuticle must be removed in order for embryos to be efficiently stained. The serosal cuticle is secreted from cells adhered to the inside of the vitelline membrane, initiating formation around 11-13h AEL [9]. Following this, the membrane appears to remain connected to the vitelline membrane through roughly 42-50h AEL, at which point the setae along the embryo pleura will be visible following removal of the serosal cuticle. If a shallow slit is created in the vitelline membrane towards the anterior end of the embryo, the anterior cap of the vitelline membrane can be removed, taking with it the attached serosal cuticle. Embryos prepared with this methodology can be extruded from their vitelline membranes free of associated serosal cuticle. Beyond the 42-50h AEL time point, the serosal cuticle becomes more strongly attached to the embryos than the vitelline membrane, particularly in older embryos that have been stored at -20°C in methanol for an extended period of time (e.g., a few months). Among these older embryos, the creation of the shallow slit inconsistently results in removal of the end of the vitelline membrane, and occasionally, extruded embryos retain the serosal cuticle in its entirety. In these cases, the serosal cuticle can be most easily removed by placement of the embryo onto a section of the double-faced tape under the PBS, followed by a firm pushing of the posterior tip of the serosal cuticle to stick it to the tape. The anterior end of the serosal cuticle can then be torn with the tungsten probe and the secured embryo can be forced out of the cuticle by pushing firmly on the posterior of the embryo until it is freed. A more comprehensive explanation of this protocol is available on *protocols.io*, along with associated video content.

Embryo developmental studies in *D*. *melanogaster* are facilitated by the established well-defined morphological staging of embryos, and continuous oviposition. Embryos from *D*. *melanogaster* can be collected over an extended period of time to obtain multiple developmental stages within a single collection since the egg follicles are continuously formed and provisioned, and eggs are continuously fertilized and laid. In contrast, egg formation in *A. aegypti* is initiated by the taking of a bloodmeal, and the follicles are provisioned collectively [10]. *A. aegypti* further produces a trypsin modulating oostatic factor hormone around 18h following the bloodmeal, which results in the provisioning of a single even-aged clutch of eggs in gravid females [11]. As a result, when gravid females are ready to oviposit, all eggs are typically fertilized and laid *en masse*. Thus, if multiple embryonic stages are needed, multiple egg chambers spanning multiple time periods of development should be established. These complicating factors highlight the need for a more detailed study of the timing and morphology of embryonic development in *A. aegypti*.

**Fig 1:**
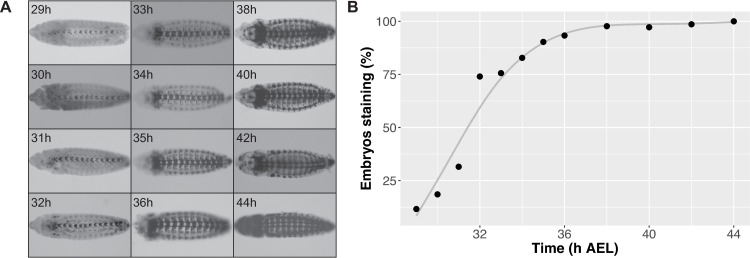
Immunohistochemical staining for acetylated α-tubulin in *A. aegypti* embryos [14]. (A) Staining for acetylated α-tubulin from the earliest time point of detection, 29h after egg laying (AEL), to the start of maximum acetylated α-tubulin staining (36h AEL); (B) Percentage of embryos staining as positive for acetylated α-tubulin. A minimum of 178 embryos was peeled for each time point (216 ± 11).

**Fig 2:**
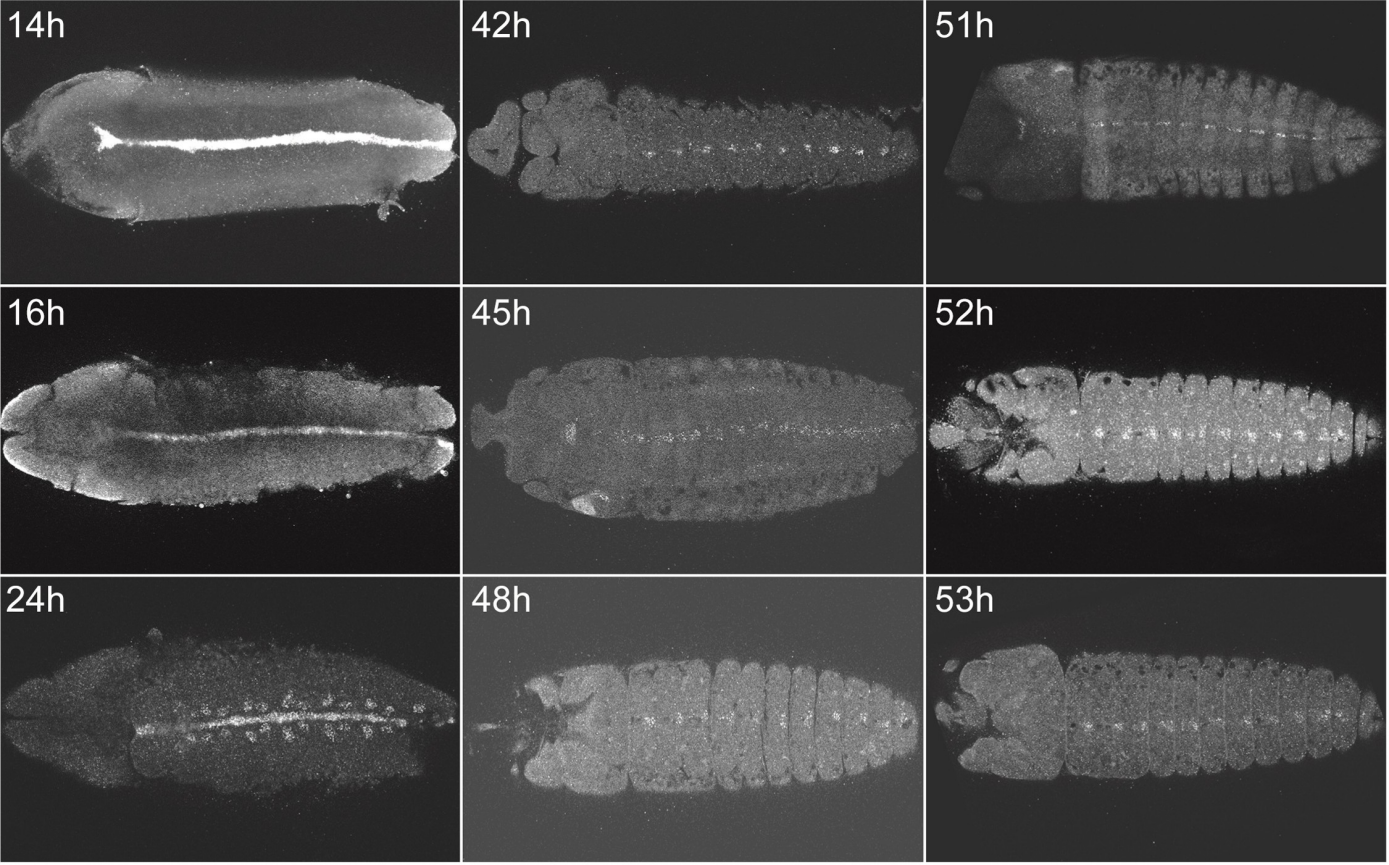
Hybridization chain reaction staining for the developmental time course for the *sim* transcript (VectorBase: AAEL011013) in *A. aegypti* from 14h to 53h after egg laying at 27°C [13].

**Fig 3:**
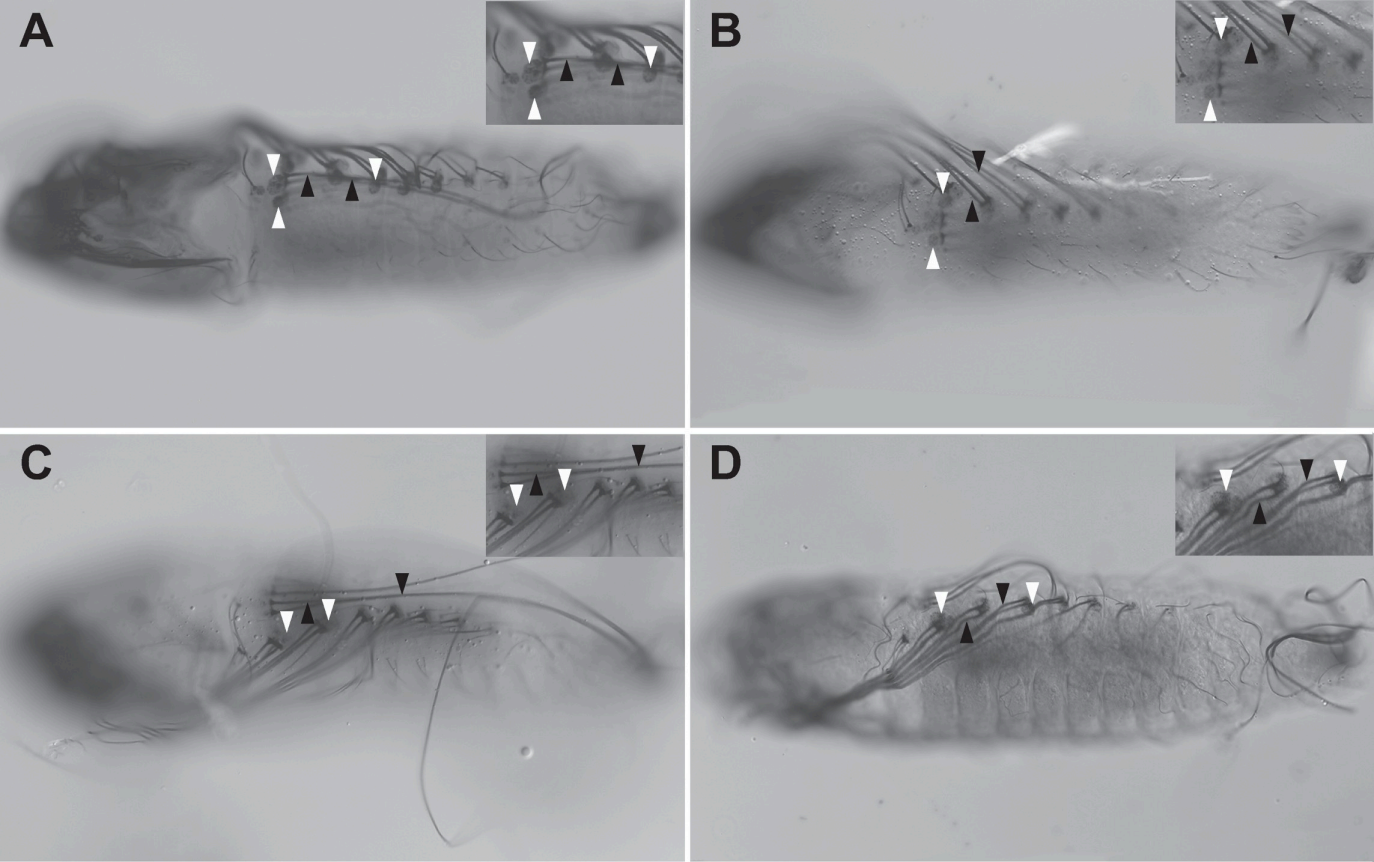
Immunohistochemical staining of wildtype 53-55h AEL *A. aegypti* embryos (Liverpool strain) showing non-specific staining of trichome cells (white arrowheads) and their associated setae (black arrowheads). Insets are zoomed images of the regions marked with arrowheads. (A) Embryo stained with rabbit anti-GFP primary antibody (Abcam, Boston, MA) and biotinylated goat-anti-rabbit secondary antibody plus streptavidin-complexed peroxidase (Vectastain ABC kit, Vector Laboratories, Newark, CA), followed by visualization using 3,3′-Diaminobenzidine (DAB). (B) Control staining using only streptavidin-complexed peroxidase, with no primary or secondary antibodies. (C) Control staining with rabbit anti-GFP primary antibody but no secondary antibody, followed by streptavidin-complexed peroxidase and DAB. (D) Control staining with biotinylated goat-anti-rabbit secondary antibody (Vectastain ABC kit, Vector Laboratories, Newark, CA) but no primary antibody, followed by streptavidin-complexed peroxidase and DAB.

Embryos prepared using our method are suitable for immunohistochemistry (Fig 1), and for in situ hybridization using the hybridization chain reaction [12] (Fig 2). We recently demonstrated that digoxigenin-labelled in situ hybridization staining in *A. aegypti* is problematic and often results in unexpected false positive signals [13]; although embryos processed using our protocol can be stained in this manner, we advise caution and extensive controls. In addition, we found that immunocytochemistry in older embryos frequently leads to false-positive staining in the abdominal sensory setae and their associated trichome cells (Fig 3). These factors need to be taken into consideration when designing and executing embryonic development studies in *A. aegypti*.

Overall, our modified protocol for the removal of the vitelline membrane of *A. aegypti* results in high-quality embryos and has been successfully tested in embryos as young as 14h AEL to as old as 55h AEL. With the capacity to peel upwards of 150 embryos within an hour, the improved protocol holds promise to expedite discovery and characterization in *A. aegypti* embryonic development studies.

## Supporting information

S1 File The protocol is provided in PDF format as S1 File and is also available from protocols.io with video accompaniment. (PDF)
