# Supplementary material for: Improved methodology for fixation and preparation of Aedes aegypti embryos
Source: PLoS One. 2024 May 31;19(5):e0304802. doi: 10.1371/journal.pone.0304802 (PMC11142583; doi:10.1371/journal.pone.0304802)
Supplement: S1 File — (PDF) [file pone.0304802.s001.pdf]

Dec 07, 2023

Protocol for fixing and peeling Aedes aegypti embryos

DOI
dx.doi.org/10.17504/protocols.io.261ged88wv47/v1

William Reid[1], Geyenna Sterling-Lentsch[1], Marc S. Halfon[1]

[1]Department of Biochemistry, Jacobs School of Medicine and Biomedical Sciences, University at Buffalo-State University of New York

Marc S. Halfon
University at Buffalo-State University of New York

OPEN ACCESS

DOI: dx.doi.org/10.17504/protocols.io.261ged88wv47/v1

Protocol Citation: William Reid, Geyenna Sterling-Lentsch, Marc S. Halfon 2023. Protocol for fixing and peeling Aedes aegypti embryos. **protocols.io** https://dx.doi.org/10.17504/protocols.io.261ged88wv47/v1

License: This is an open access protocol distributed under the terms of the **Creative Commons Attribution License**, which permits unrestricted use, distribution, and reproduction in any medium, provided the original author and source are credited

Protocol status: Working
We use this protocol and it's working

Created: December 01, 2023

Last Modified: May 20, 2024

Protocol Integer ID: 91954

Funders Acknowledgement:
National Science Foundation
Grant ID: IOS-1911723

Abstract

The collection, fixing, and immunohistochemical staining of Aedes aegypti embryos is challenging in comparison to D. melanogaster since the vitelline membrane of Ae. aegypti must be manually removed. Herein we report on an improvement for the methods to prepare Ae. aegypti embryos. The adapted protocol increases the throughput capacity of embryos by an individual user, with experienced users able to process an average of 100-150 embryos per hour. The protocol provides high-quality intact embryos that can be used for morphological, immunohistochemical, and in situ RNA hybridization studies. Critical to the success of the optimized protocol is the selection and description of the tools required, and differing approaches for younger and older embryos.

Attachments

DOCX

n7sicat3x.docx

35KB

Materials

Materials:

A	B	C
Item	Optional/Recommended vendor	Part number
Embryo collection		
Aspirator		
Whatman #1 filter paper		
50 mL conical tubes		
Plastic shoe box 13 x 8 x 5”		
6 cm-diam Petri dishes		
Aluminum foil		
Distilled water		
Defibrinated sheep blood	Hardy Diagnostics	SB100
ATP		CAS 56-65-5
Embryo fixing		
Egg basket (100 µm cell strainer)	VWR	76327-102
20 mL Glass scintillation vials		
Pasteur pipettes		
Heptane		
Methanol		
PBS(P) buffer		
PT buffer		
1.5 mL polypropylene tubes		
Petri dishes		
Bleach (6% NaClO)		
Distilled water		
16% methanol-free formaldehyde	Polysciences	18814-20
PEM buffer		
Fine spotter paint brush	Princeton Artist Brush Co	3750MSP-20/0
-70°C freezer (optional)		
Squirt bottle (distilled water)		
60°C incubator		
Embryo peeling		
0.05” stab knife	Fine Science Tools	72-1551

A	B	C
Tungsten wire probe		
Roboz microprobe	Roboz	RS-9420
Glass well slide		
Glass coverslips (22 x 22mm)		
Plastic coverslips (22 x 22 mm)		
Double faced tape (¹⁄₄")	3M brand	S-15941
Fine spotter paint brush	Princeton Artist Brush Co	3750MSP-20/0
Glass plate		
1.5 mL polypropylene tubes		
Scissors		
Forceps		
Tools		
10 mL serological pipette		
Rubber tubing (1/4” ID; 3/8” OD)	Tygon	E-3603 TET-250B
Chiffon fabric		
0.005” tungsten wire	Ted Pella, Inc	27-Nov
In-line HEPA filter	Bugdorm	DP1009
Inoculating loop holder	Fine Science Tools	26018-17
9V battery		
1M KOH (or NaOH)		
9V battery housing	Jameco Electronics	216427
Glass slide (dimensions)		
Aquarium sealant	Dow Corning	DOWSIL‰ 732 Multi-Purpose Sealant
5 mL syringe		

Equipment:

- Stereo dissecting microscope
- 60°C incubator
- 4°C refrigerator
- -20°C freezer
- -70°C freezer

PBS(P)

A	B	C
Reagent	Quantity (for 1 L)	Final concentration (10X)
NaH2PO4	2.56 g	18.6 mM
Na2HPO4	11.94 g	84.1 mM

A	B	C
NaCl	102.2 g	1.75 M

PEM Buffer

A	B
PIPES (pH 6.95)	0.1 M
EGTA	2 mM
MgSO4	1 mM

5xPEM Buffer

A	B
PIPES (pH 6.95)	0.5 M
EGTA	10 mM
MgSO4	5 mM

PEM-F

A	B
Reagent	Volume (for 10 mL)
1X PEM buffer	7.7 mL
16% Formaldehyde (methanol-free)	2.3 mL (final concentration 3.68%)
Prepare PEM-F fresh just before use.	

PT

A	B
ddH2O	44.5 mL
PBS(P) (10X)	5 mL
10%(w/v) Tween 20	0.5 mL
Store at 4°C or room temperature.	

Blood, Sheep, Defibrinated, Screw Cap Bottle, 100ml **Hardy Diagnostics Catalog #**SB100

Formaldehyde, 16%, methanol free, Ultra Pure **Polysciences, Inc. Catalog #**18814-20

Nickel Plated Pin Holder **Fine Science Tools Catalog #**26018-17

9V Battery Holder with 6 Inch Wires **Jameco Electronics Catalog #**216427

Safety warnings

This protocol contains hazardous chemicals including formaldehyde, heptane, methanol, and animal blood. Appropriate PPE and institutional-specific safety precautions should be taken. (PPE was occasionally removed during production of the videos to improve visualization.)

Before start

This protocol was developed using the Liverpool strain of Ae. aegypti reared under optimal conditions at 27°C, 80% RH and a 12:12 (L:D) photoperiod. Larvae were reared in 14 x 8 x 5 " containers and fed a slurry of Tetramin fish food (Spectrum Brands Pet, LLC, Blacksburg, VA) ad libitum. Adults were provided with raisins as a sugar source.

Preparation of an aspirator

1

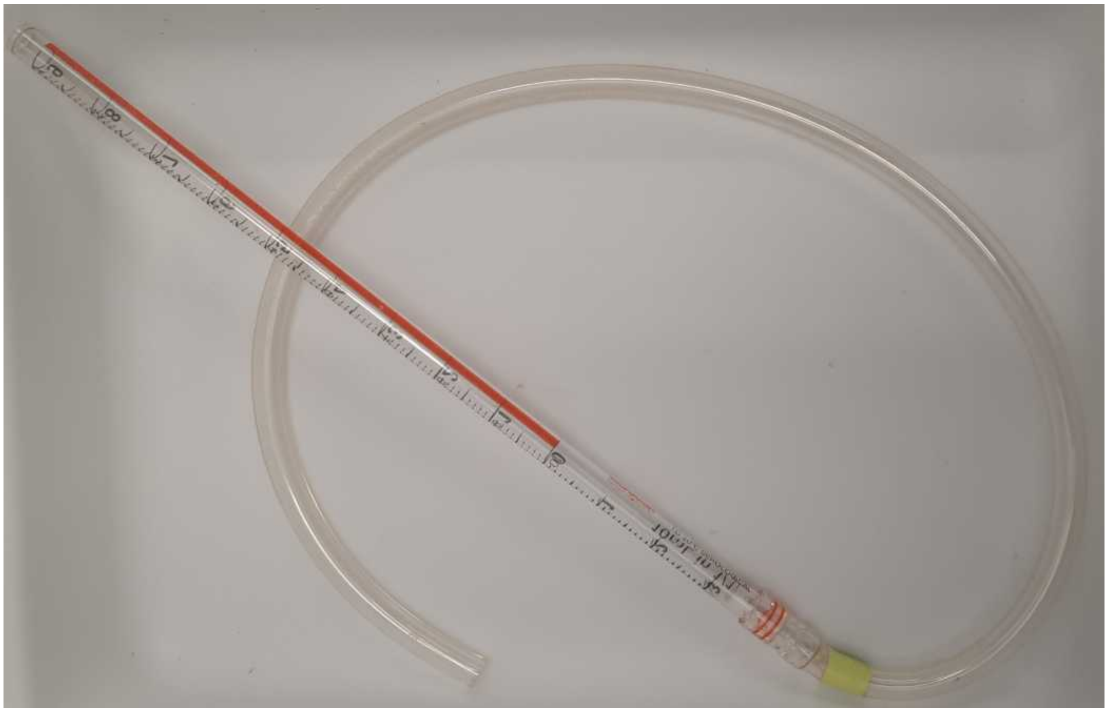

Figure 1. Aspirator tool for collecting mosquitoes.

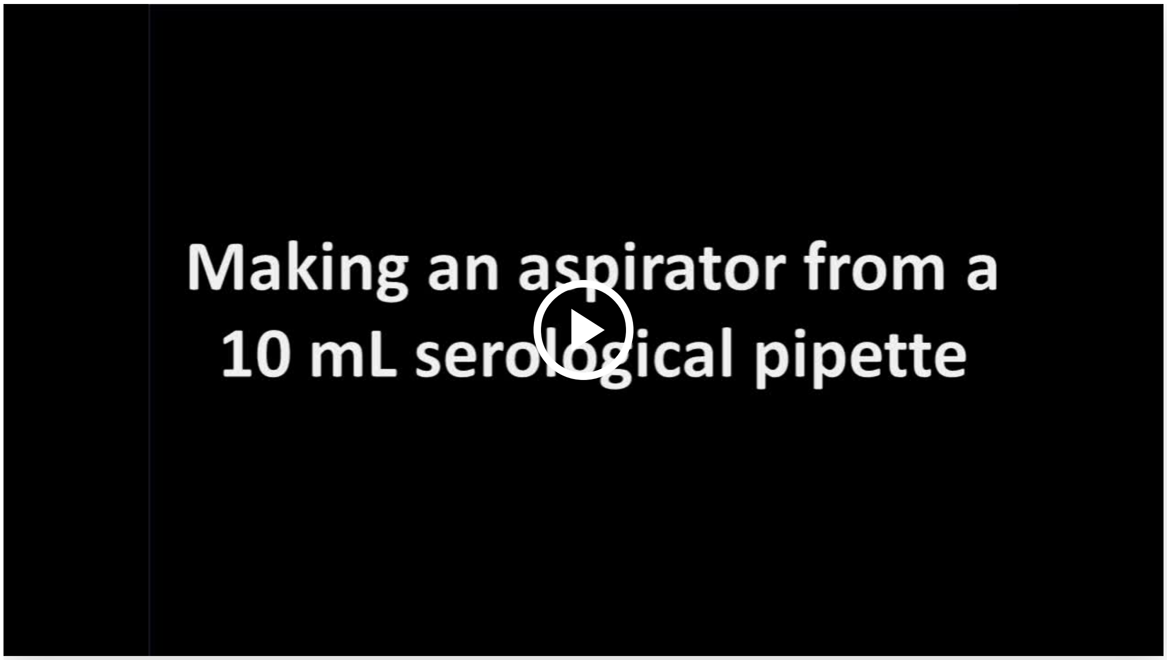

Cut both ends from a 10 mL serological pipette. Save the end with the cotton plug.

2 Trim the tapered tip and remove any sharp edges.

3 Whittle the thinner plastic end of the serological pipette where the cotton plug is.

4 Fix two layers of chiffon fabric as a blocking filter by inserting the shaved end of the pipette tip into the pipette.

5 Attach a length of tygon tubing for mouth aspiration.

Note

The tubing is easier to apply if it is first warmed in a hot water bath (near boiling). Further, an inline HEPA filter can be added to the tubing to prevent the inhalation of scales.

Preparation of an egging chamber

6

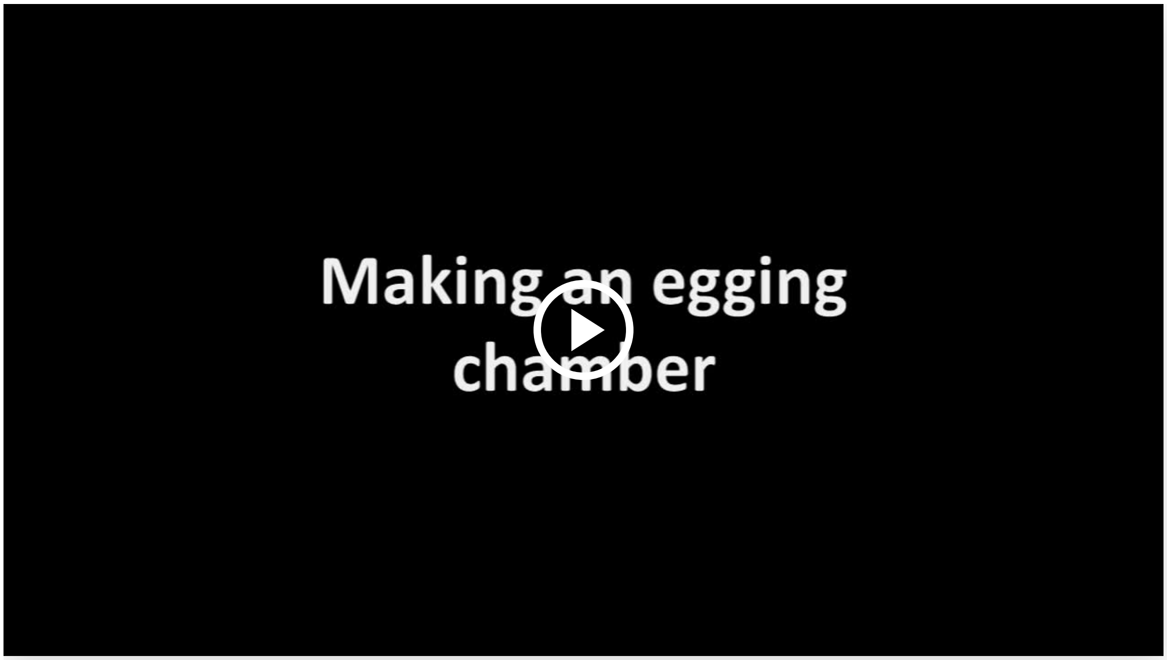

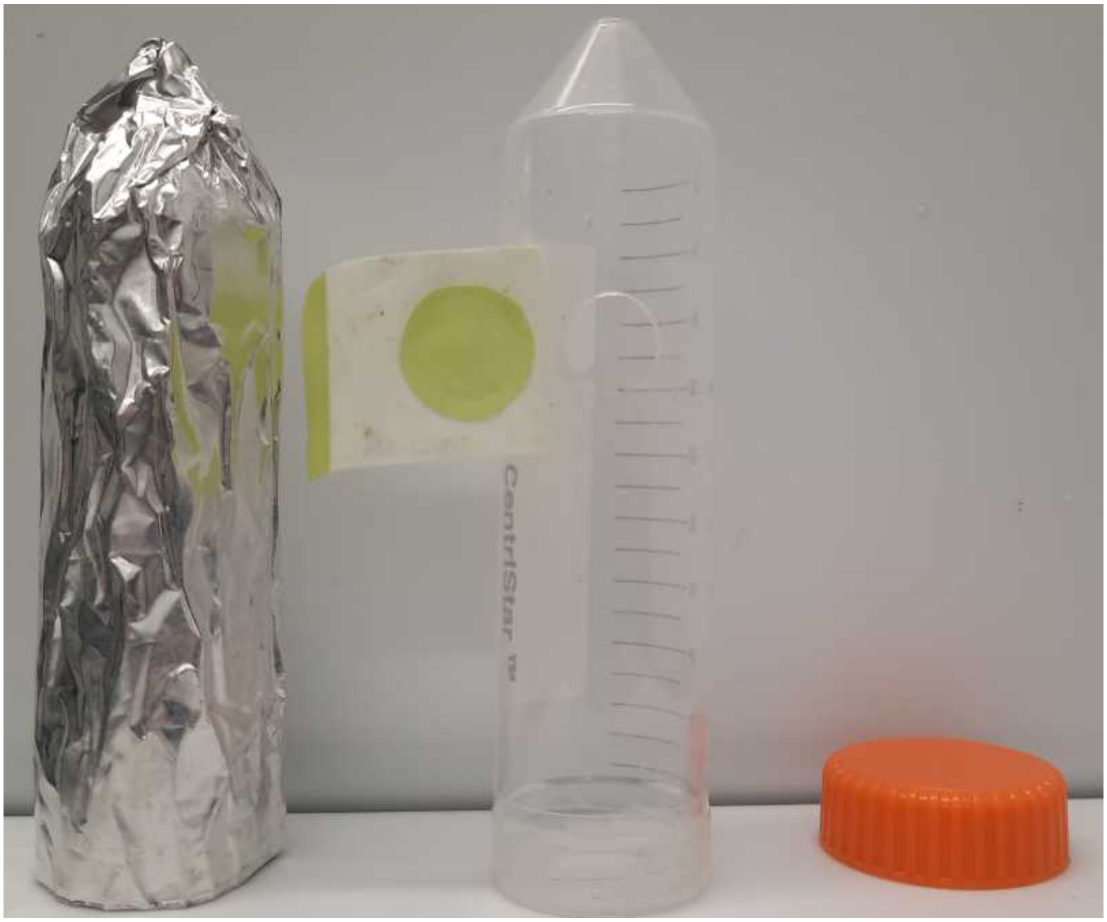

Figure 2. Chamber for egg collection.

Remove the threads from the cap and tube of a 50 mL conical tube.

Note

The threads need to be removed so that the paper that will be placed for egg laying remains in tact when the lid is added/removed.

7 Create an entry port for mosquitoes by drilling a hole that is slightly larger than the outer diameter of a 10 mL serological pipette into the 50 mL conical tube.

8 Use a strip of laboratory tape to create a removable closure over the entry port.

9 Place a second smaller section of tape on the back of the tape closure so that when the mosquito entry port is closed, there is no sticky side of the tape facing the inside of the 50 mL conical tube.

Note

Alternatively two layers of dental dam with orthogonal slits can be taped over the entry port.

Electrosharpen a tungsten wire

10

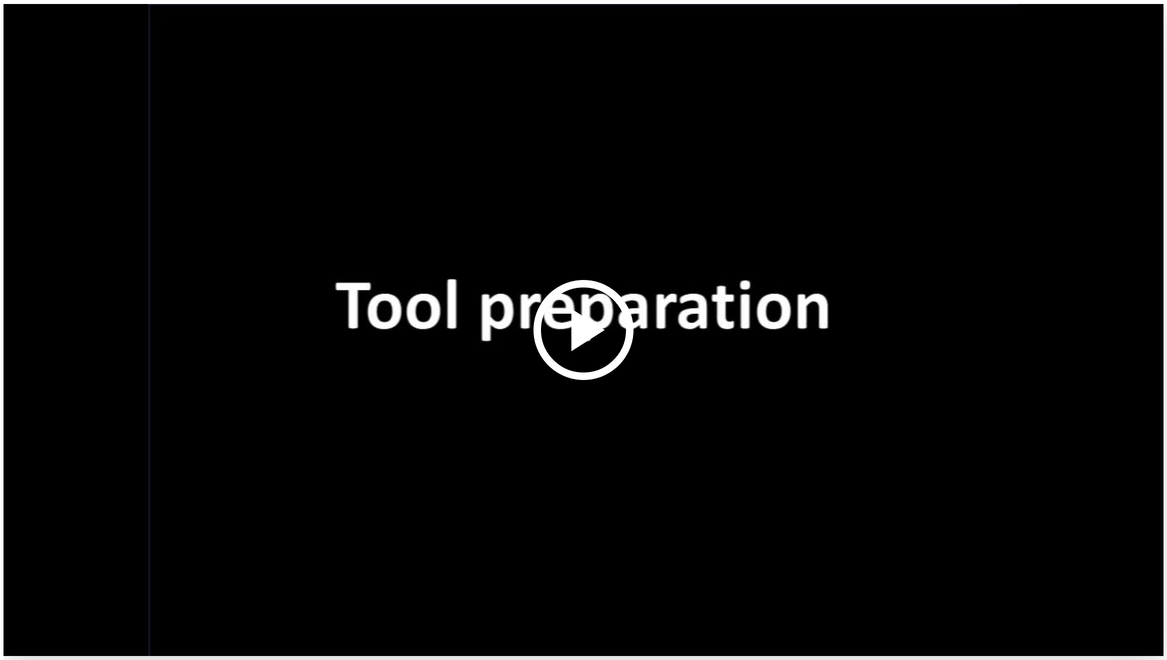

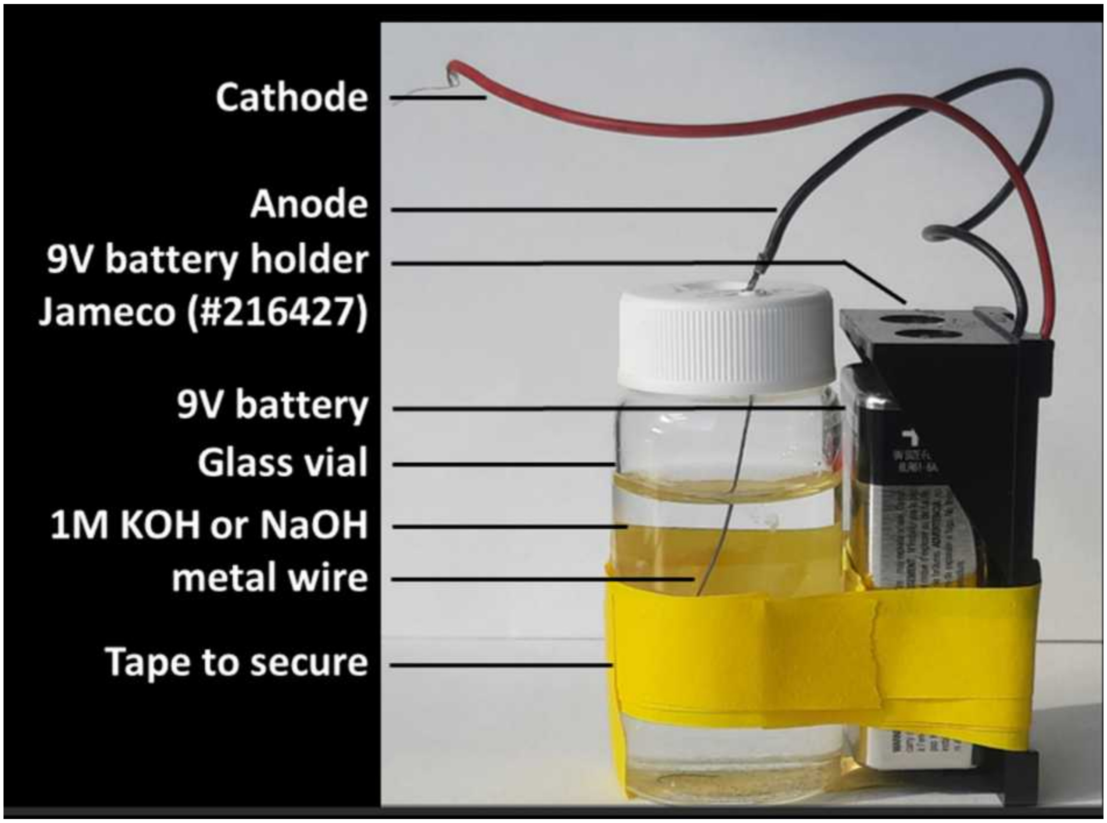

Figure 3. Set up for electrosharpening a tungsten wire.

Cut a 3-4 cm length of 0.005” tungsten wire.

11 Secure the tungsten wire in an inoculating loop holder.

12 Connect the anode (negative terminal) of a 9V battery to a 1.0M solution of KOH or NaOH.

13 Connect the cathode (positive terminal) of the 9V battery to the metallic end of the inoculating loop holder.

14 Repeatedly dip the end of the tungsten wire into the 1.0M solution of KOH (or NaOH) to electrosharpen the tungsten wire.

Note

Periodically check the tungsten wire under a stereoscope to determine if additional Electrosharpening is needed.

Collection of embryos

1h

15 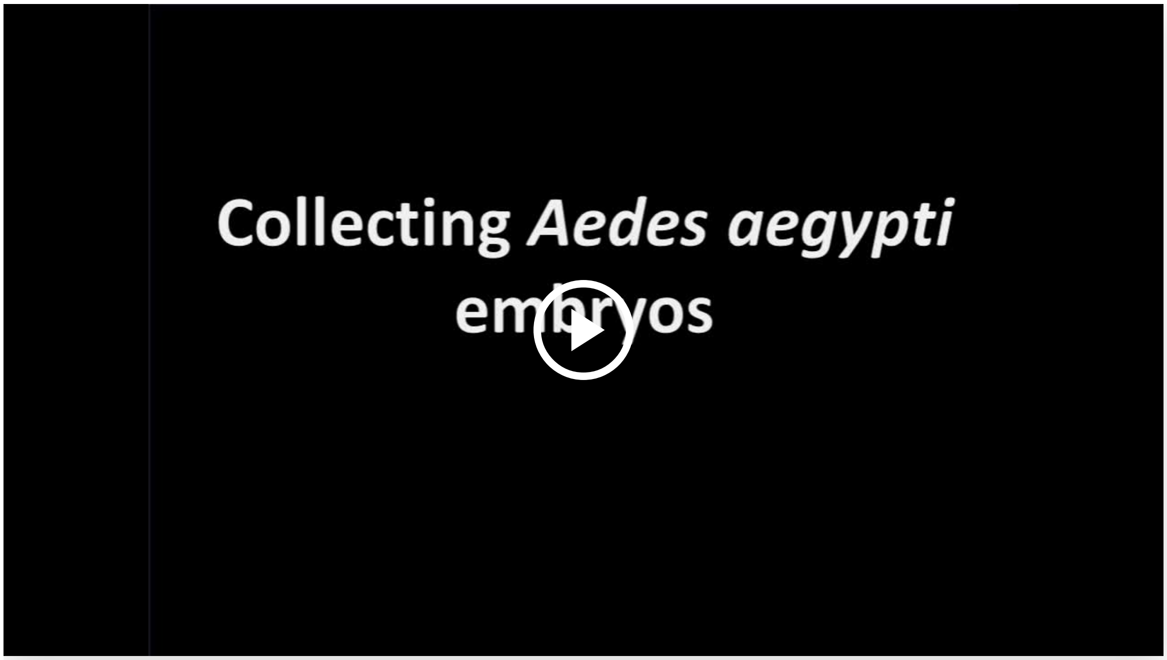

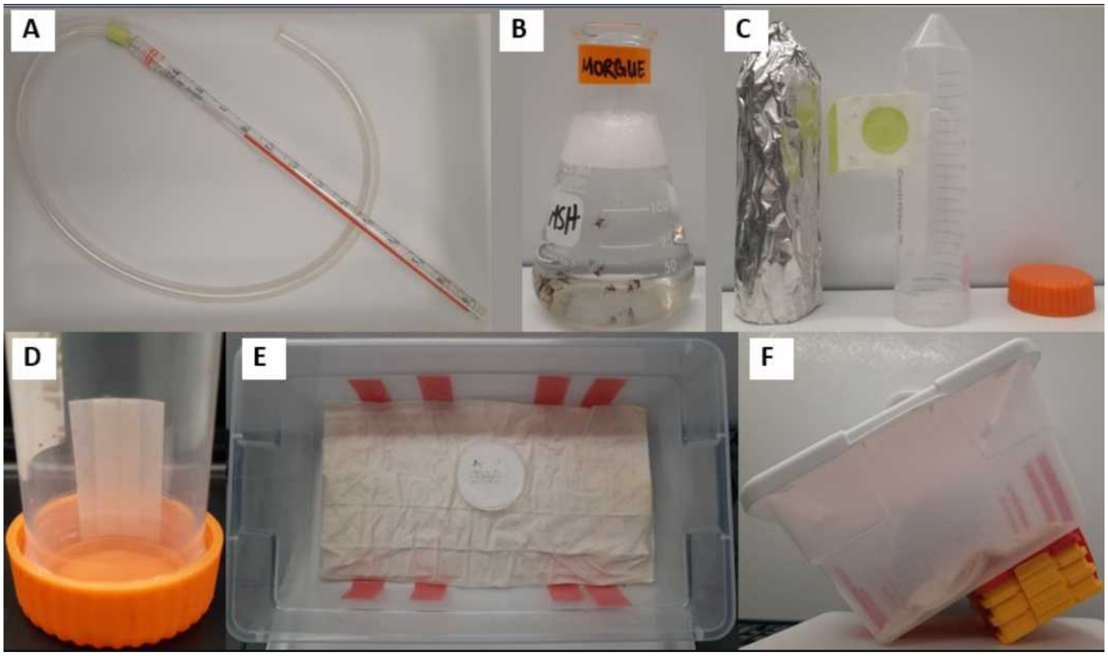

Figure 4. Collection of Ae. aegypti embryos. (A) Aspirator to collect females; (B) Soapy water morgue for killing males; (C) Egging chamber to allow females to lay eggs; (D) Overlapping of filter paper strips for oviposition; (E) 6-cm Petri dish for ageing embryos; (F) Shoe box humid chamber to maintain humidity during embryo ageing.

Place 250-300 Ae. aegypti pupae into a cage at a sex ratio ranging from 1:1 to 2:1 (F:M).

16 Provide the mosquitoes with a fresh blood meal three to five days post adult emergence.

Note

When using a non-live blood source (e.g., a Hemotek feeder), the addition of ATP to the blood at a final concentration of 10 µM encourages females to take a full meal.

17 Four days post blood meal, use the aspirator to transfer 20-50 females into an egging chamber lined with three overlapping strips of wetted Whatman #1 filter paper.

Note

On the third day post blood meal, aspirate the males from the cage and kill in soapy water. Males can be visually identified by their plumose antennae. Replace any open water source for the mosquitoes with clean water since females will lay eggs on any dead mosquitoes floating on the water. Alternatively, the water cup can be removed entirely, but do not deprive mosquitoes of water for longer than 10-12h. The 20-50 females can be collected in a single aspiration.

18 Darken the conical tube by covering the tube with aluminum foil. Allow the mosquitoes to lay eggs undisturbed for 01:00:00 . 1h

19 Transfer the egg paper to a plastic petri dish lid lined with two layers of wetted Whatman #1 filter paper and close the lid with the bottom of the dish.

20 Incubate the embryos at 27 °C for the desired duration of time. Make sure the embryos are positioned on a slight incline so that they are not submerged in water.

Fixation of embryos

36m

21

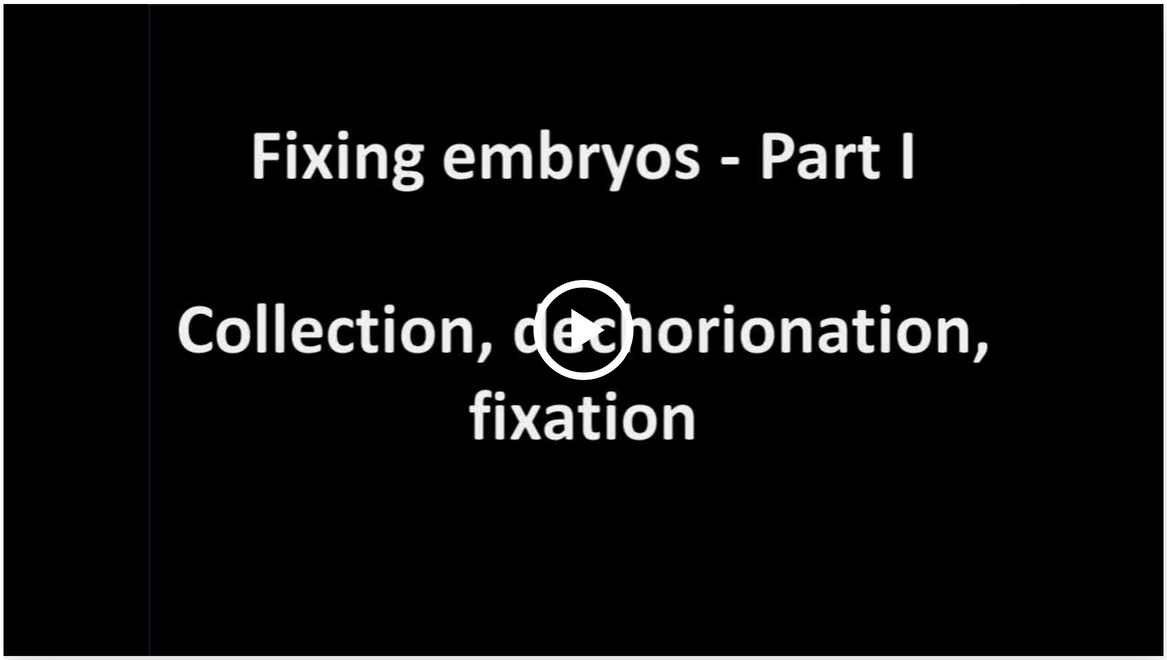
Fixing embryos - Part I
Collection, dechorionation,
fixation

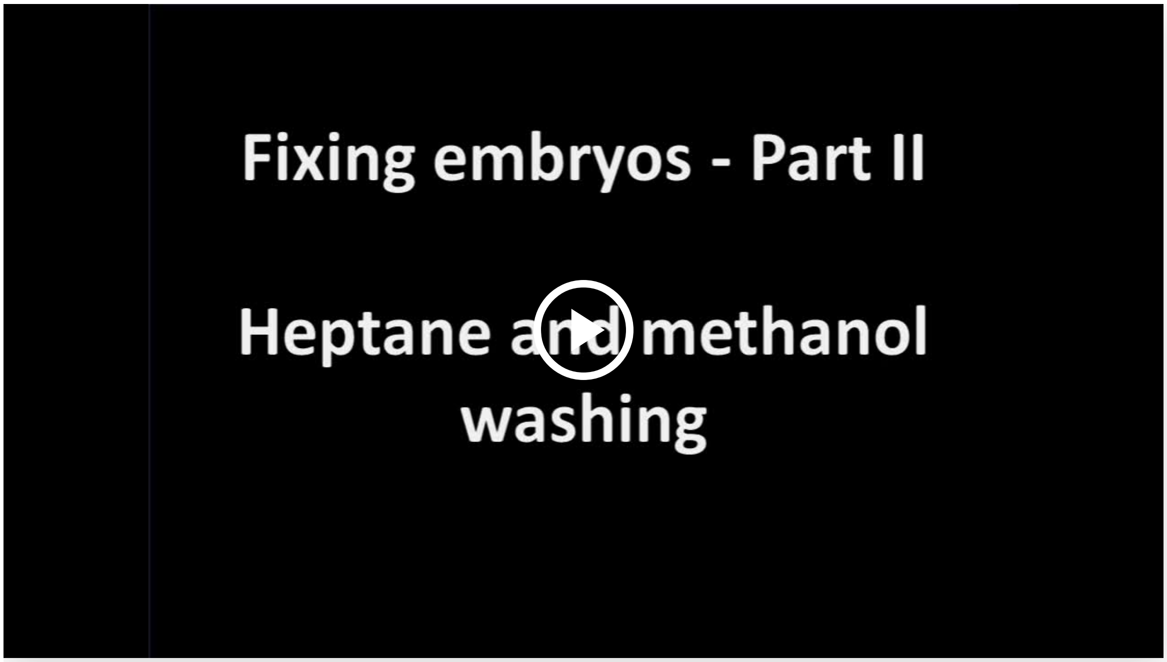
Fixing embryos - Part II
Heptane and methanol
washing

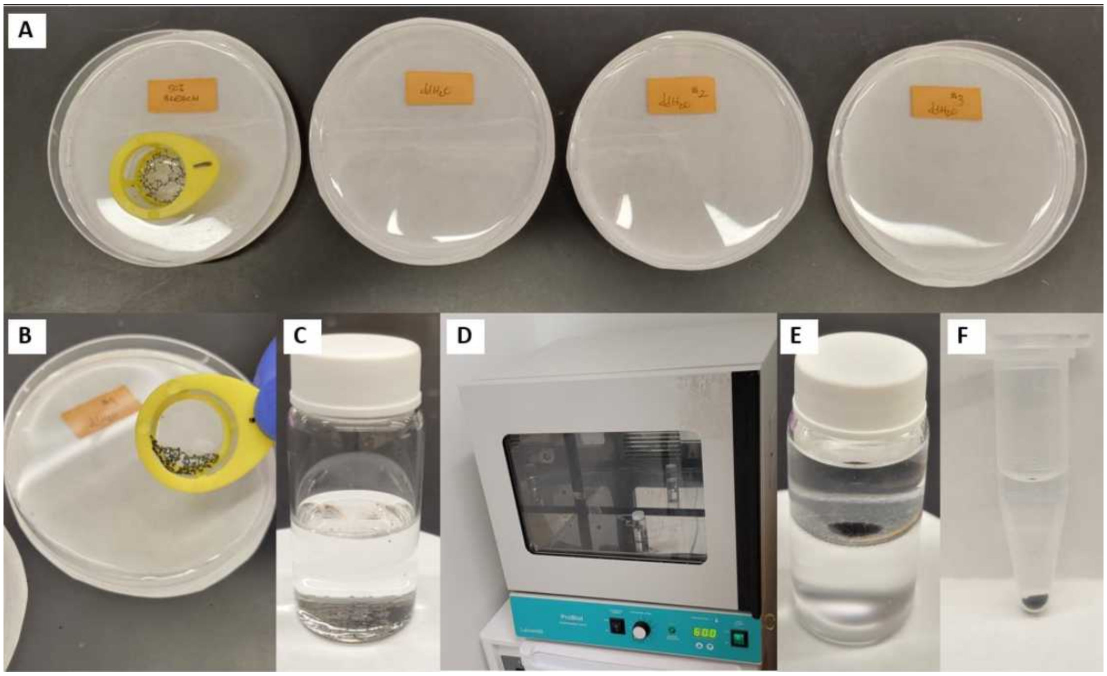

Figure 5. Supplies and equipment needed for the fixation of Ae. aegypti embryos. (A) Bleach and rinsing station for dechorionating embryos; (B) Embryos rinsed to the inside edge of a 100 µM cell strainer for easier transfer to a glass scintillation vial; (C) 20 mL glass scintillation vial with embryos in PEM-F fixing solution; (D) Embryos being fixed at 60°C in PEM-F fixative; (E) Fixed embryos partitioned over heptane and methanol; (F) Embryos washed with methanol for storage at -20°C.

Prepare the following solutions for fixing embryos: 20 mL 50% bleach (Final NaClO = 3%), 10 mL PEM-F (prepared fresh), 10 mL MeOH, 10 mL heptane.

22 Set up an egg basket (i.e., 100 µm cell strainer) in a 9-cm-diam Petri dish bottom and fill the dish bottom with distilled water.

23 Dislodge the embryos from the edges of the filter papers with a fine tipped paintbrush.

24 Rinse embryos into the egg basket with distilled water.

25 Dechorionate embryos in 50% bleach (3% NaClO) for 00:00:30 . 30s

Note

The embryos will float in the bleach. Use a Pasteur pipette to rinse the embryos with bleach during the dechorionation to ensure that they are fully dechorionated.

26 Generously rinse embryos in distilled water three times.

> **Note**
>
> The embryos need to be rinsed profusely to remove any residual bleach. In addition, rinse the egg basket with water to make sure that residual bleach is not present.

27 Rinse embryos from the egg basket into a scintillation vial.

28 Remove excess water from the scintillation vial and add 10 mL of PEM-F.

> **Note**
>
> Some embryos will float on the PEM-F. Embryos can be submerged in the PEM-F by dropping droplets of PEM-F on them until they sink.

29 Incubate embryos in PEM-F at 60 °C for 00:30:00 . 30m

30 Remove the PEM-F and add 10 mL of heptane.

> **Note**
>
> It is important to remove all of the PEM-F before adding the heptane. In addition, the embryos should swirl freely in the heptane, otherwise they will not stick to the tape during the manual peeling step. If the embryos stick to the scintillation vial, add 10 mL of methanol and shake the embryos for 30 seconds. Remove the heptane and methanol, then add 10 mL of fresh heptane. If the embryos now swirl freely, proceed to step 17, otherwise repeat the methanol wash and the repeat addition of the heptane.

31 Transfer embryos to a -70 °C freezer for 00:05:00 . 5m

32 Add 10 mL methanol and shake the vial under running hot (60 °C) water for 00:00:30 . 30s

> **Note**
>
> The freezer/running hot water steps are carried over from Clemons et al. (2010). In our experience, steps 31 and 32 can be conducted at room temperature with no detrimental effects.

33 Remove the upper heptane layer and most of the methanol from the embryos.

Note

Let the scintillation vial stand for 5 minutes to allow the heptane to separate from the methanol. The embryos will collect into a group at both the bottom of the methanol layer and at the heptane: methanol interface. When enough of the heptane layer has been removed, the embryos at the heptane:methanol interface will sink and combine with the embryos grouped at the bottom of the methanol layer.

34 Add an additional 10 mL methanol to the scintillation vial.

Note

The embryos can be collected into a pile by swirling the scintillation vial.

35 Transfer embryos to a 1.5 mL tube.

36 Wash embryos with three changes of 1 mL of 100% methanol. Embryos can be stored in methanol at -20 °C . Alternatively proceed with the rehydration step.

Note

The vitelline membrane of younger embryos (<20h old after egg laying at 27°C) can be difficult to remove if embryos are maintained under methanol for extended periods of time. We recommend that younger embryos be rehydrated following the methanol washing steps and the vitelline membrane removed within a few days.

Removal of the vitelline membrane (“embryo peeling”)

30s

37

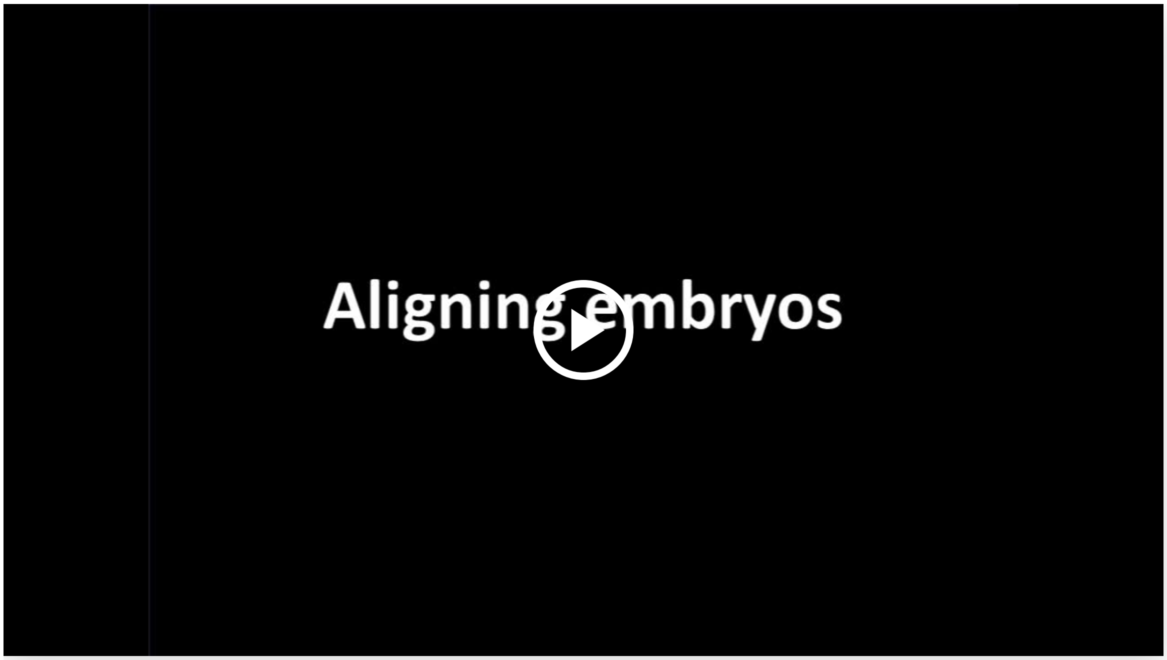

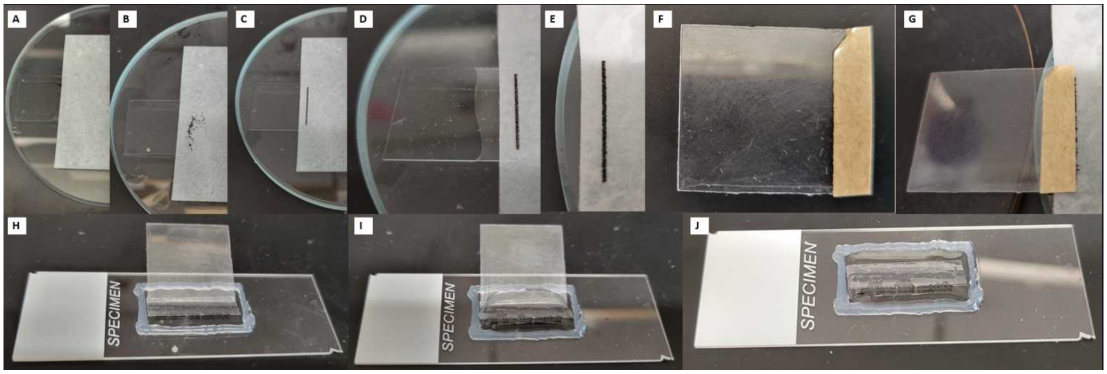

Figure 6. Alignment of embryos for removal of the vitelline membrane. (A) Glass plate with a strip of wetted filter paper overlayed with a glass coverslip; (B) Fixed embryos transferred to the wetted filter paper; (C) Embryos aligned against the edge of the glass coverslip with the anterior ends to the right; (D) Drying of the filter paper to 'break' the PBS connection between the glass coverslip and the filter paper; (E) Embryos shifted to the edge of the glass plate to facilitate embryo transfer to the double faced tape; (F) Strip of double faced tape affixed to the edge of a 22mm plastic coverslip with the top edge peeled back for easier removal of the protective paper cover; (G) transfer of the embryos to the edge of the double faced tape; (H) Transfer of the double faced tape to a well slide with the embryos top-side up;(I) Flooding of the well with PBS; (J) The final well slide with PBS-submerged embryos and the plastic coverslip removed.

Rehydrate the embryos using a methanol/PBS(P) dilution series (1 mL and 10 min each): 75%, 50%, 25%, 100% PBS(P), PT.

Note

Add the methanol/PBS(P) solutions slowly so that the embryos will rehydrate under the solutions.

38 Replace the final rehydration with a new 1 mL aliquot of PT and store embryos Overnight at 4 °C prior to peeling.

30s

Note

The embryos can be peeled immediately following rehydration; however they tend to peel easier if they are allowed to soak Overnight at 4 °C in PT buffer.

39 Wet a 1”x2” square of Whatman #1 filter paper with PBS(P) and place on a smooth glass surface.

40 Add a glass coverslip so that it slightly overlaps onto the filter paper.

Note

Make sure that there are no air bubbles underneath the coverslip.

41 Flood the underneath of the coverslip with PBS(P) so that a complete “water bridge” is formed. Wick off any excess PBS(P).

42 Affix an equal-length strip of double-faced tape to the edge (1-2 mm overlapping) of a plastic coverslip.

43 Trim the other edge of the double-faced tape to leave a clean edge.

44 Peel back a corner of the tape backing and place the prepared transfer tape aside.

45 Add 50-100 fixed embryos along the edge of the glass coverslide overlapping the wetted filter paper.

46 Gently brush embryos to the edge glass coverslip.

Note

Discard broken or misshapen embryos. Add and remove PBS(P) as needed to maintain a level of wetness so that the eggs remain wet, but the paper is dry enough to be able to move an egg without it floating out of position.

47 Use a fine tipped paintbrush to position the posterior (thinner end) of the embryo towards the edge of the glass slide and the anterior (wider end) away from the glass slide.

48 Continue to align embryos tightly against each other until all embryos have been aligned.

Note

The embryos should be touching each other in the alignment.

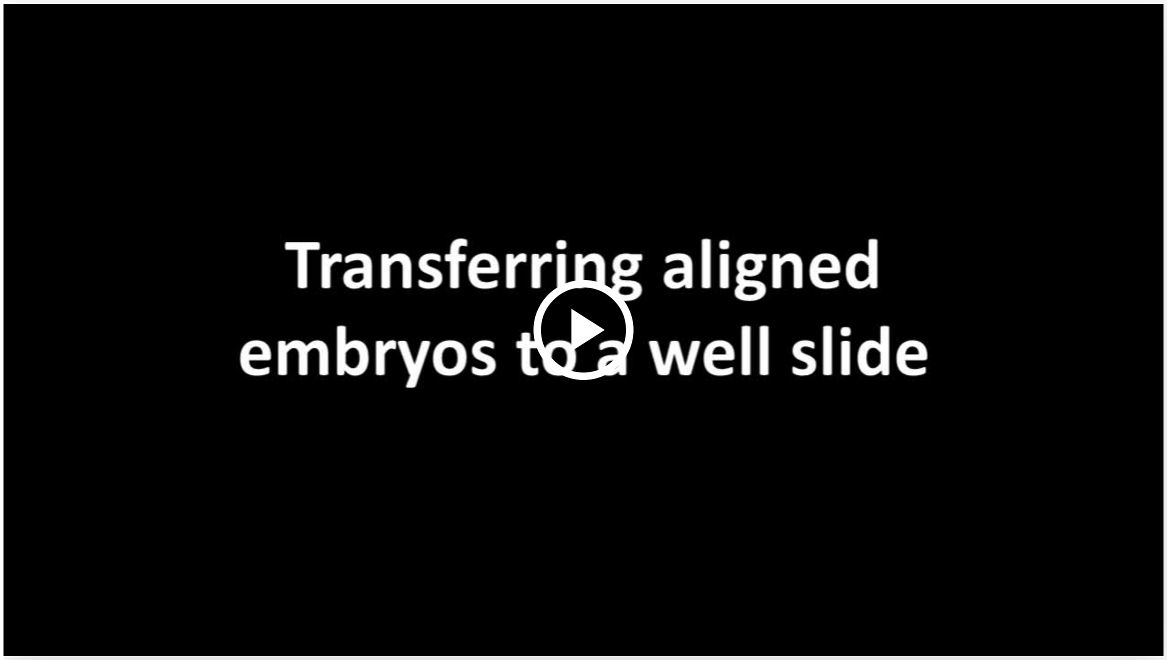

49 Remove the PBS from underneath the glass coverslip by pressing paper toweling onto the filter paper.

50 Remove the glass coverslip and gently move the line of embryos towards the edge of the glass plate by sliding the paper across the glass surface without lifting.

Note

Alternatively, the tape can be added to the embryos without moving the paper, however we find it easier to properly overlay the tape if the filter paper is moved to the edge of the glass surface.

51 Transfer the embryos to the double faced tape so that the anterior of the embryos slightly overhangs the outer edge of the tape. Gently press on the backing of the double faced tape to ensure that the embryos are affixed to the tape.

Note

It is important to press gently so that the embryos are not crushed during the transfer

52 Remove the tape backing and place the tape into the well of a glass well slide (embryo side up).

53 Flood the well with PBS(P). Add PBS(P) as needed to prevent the embryos from drying out.

Peeling older embryos (>20h AEL at 27°C)

54

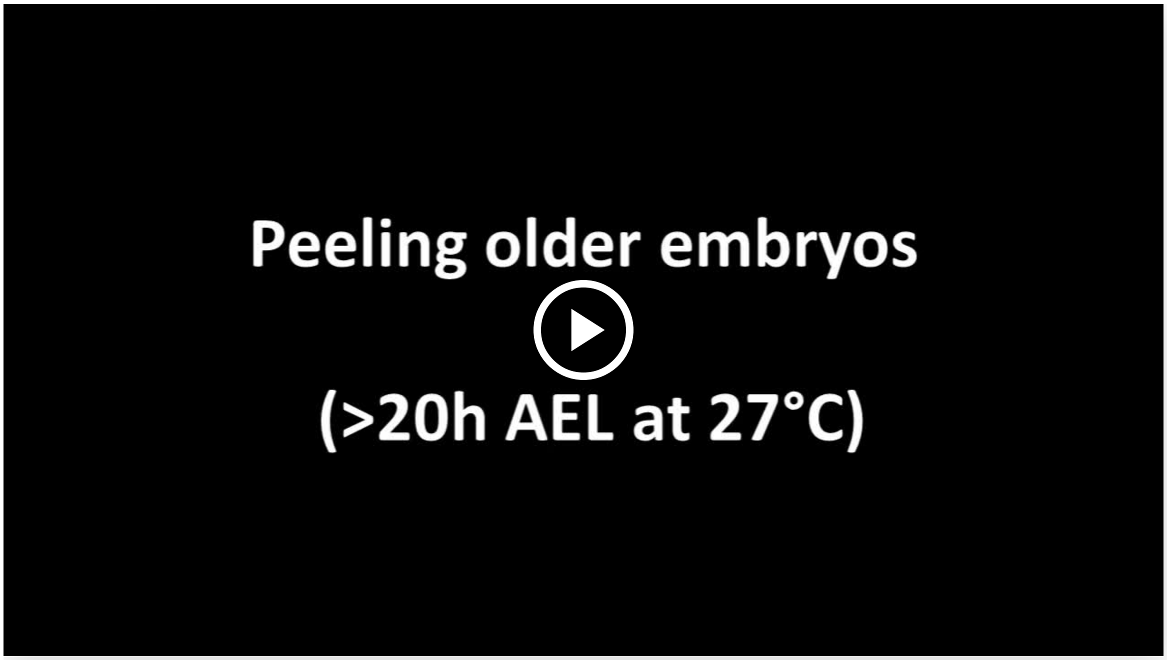

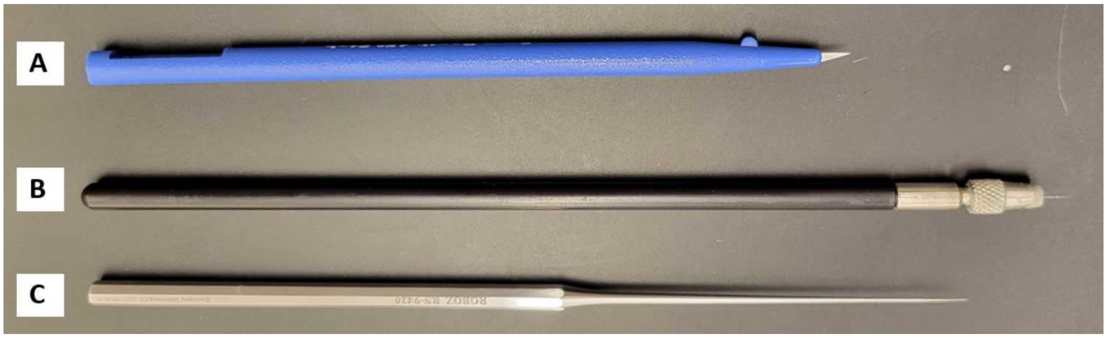

Figure 7. Tools used for removal of the vitelline membrane. (A) Stab knife for the creation of a slit towards the anterior end of the embryo; (B) Sharpened tungsten wire probe for removal of the anterior end of the embryo; (C) Roboz tool for extrusion of the embryo following removal of the anterior end of the embryo.

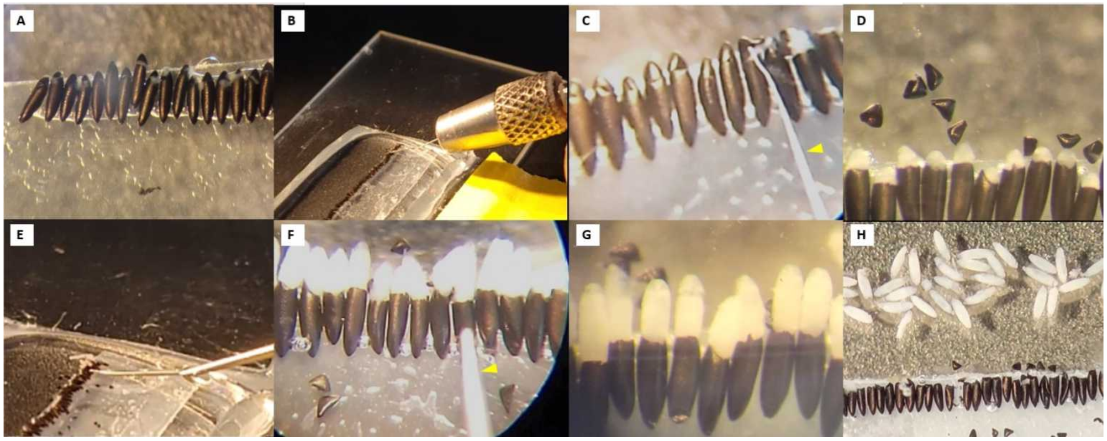

Figure 8. Intermediate and final steps in the removal of the vitelline membrane for older embryos. (A) Aligned embryos with a small opening in the vitelline membrane at the anterior end made by the stab knife; (B,C,D) Removal of the anterior end "cap" of the embryo with a tungsten wire probe; (E,F,G) Extrusion of embryos from the vitelline membrane using the Roboz tool; (H) Peeled embryos ready for transfer to a 1.5 mL tube. The yellow arrows indicate the wire probe and Roboz tool in panels (C) and (F), respectively.

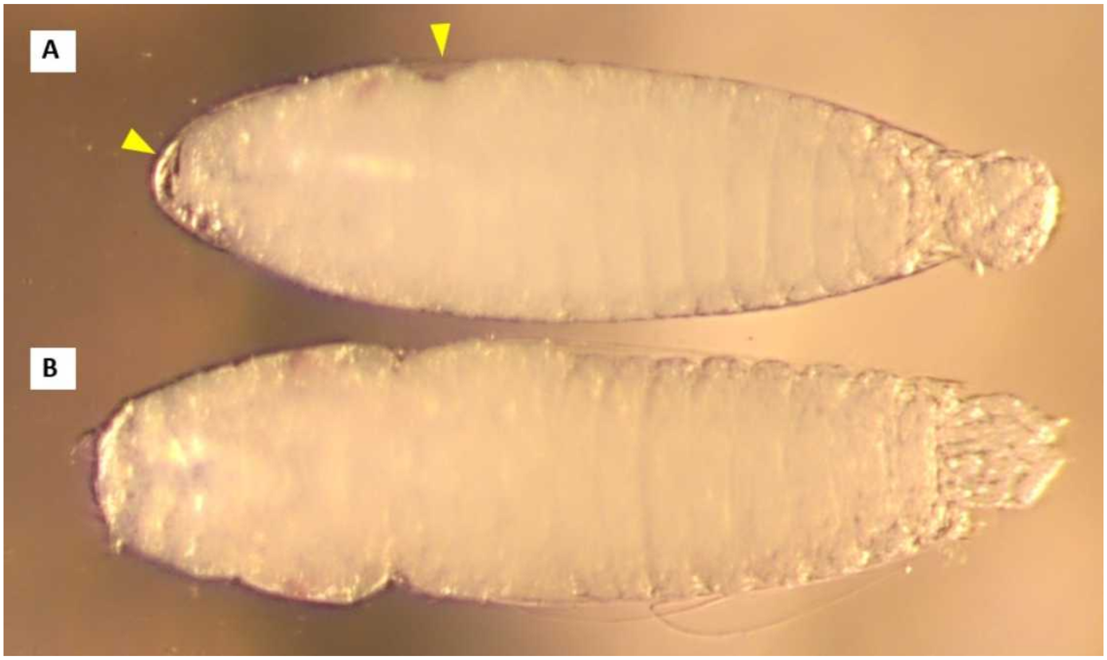

Figure 9. The serosal cuticle retained on older embryos (52h AEL at 27°C). (A) 52h AEL embryo retaining the serosal cuticle indicated by the yellow arrows; (B) 52h AEL embryo free of the serosal cuticle.

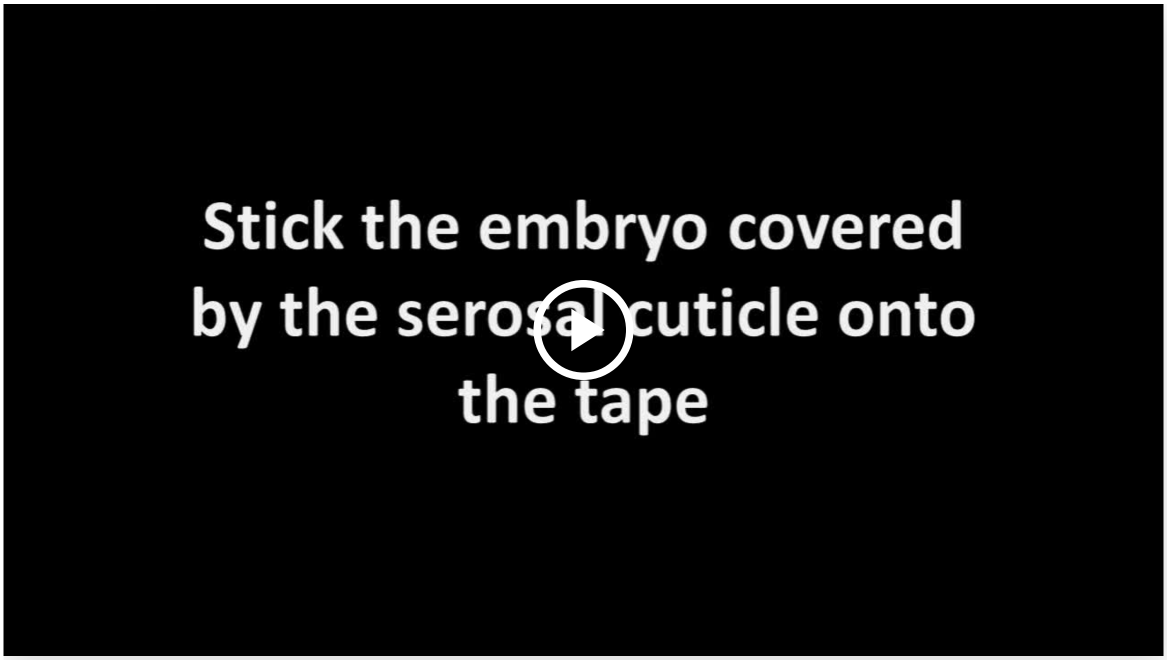

Make a small incision at the anterior of each embryo using a fine stab knife.

Note

The poke or cut should not be deep into the embryo and only needs to be large enough for the insertion of a fine tungsten wire probe.

55 Carefully slip the anterior end of the vitelline membrane off of the embryos by gently inserting the tungsten wire underneath the membrane at the hole/seam and pushing forward with a very slight upward pressure. The main pressure of the tungsten wire should be forward with a very slight upward pressure.

Note

If the tip of the anterior end tears, use the tungsten wire to tuck the vitelline membrane into the tape glue.

56 Remove the anterior end of the vitelline membranes from all of the embryos.

Note

By first removing the anterior end of the vitelline membranes from all of the embryos prior to extrusion, the cavity of the vitelline membrane is able to fill with PBS(P), making the embryos easier to extrude.

57 Rinse the loose vitelline membrane fragments from the slide with a gentle stream of PBS(P), then refill the reservoir with clean PBS(P).

Note

This step is optional, however it will make it easier to collect the embryos without collecting fragments of the vitelline membrane.

58 Using the blunt Roboz probe, gently push on the posterior end of the embryo until it extrudes out of the membrane.

Note

When gently pushing on the posterior of the embryos to extrude the embryo, stop if the embryo no longer easily extrudes. Allow for the cavity of the vitelline membrane to refill with PBS(P), before continuing to push on the posterior of the embryo. We recommend partially extruding all embryos first, to allow for the cavities of the vitelline membranes to refill with PBS(P) before fully extruding all embryos.

59 Move and press any extruded embryos retaining a serosal cuticle into the sticky surface of the double faced tape.

60 Tear the serosal cuticle at the anterior end of the embryo.

61 Extrude the embryo from the serosal cuticle by pushing on the posterior end of the embryo with the blunt side of the tungsten wire probe.

Note

The serosal cuticle on embryos younger than 50-60h AEL at 27°C typically remains with the vitelline membrane and does not need to be manually removed following embryo extrusion from the vitelline membrane.

62 Collect embryos into a pile and transfer into a 1.5 mL tube using a Pasteur pipette.

Note

Wet the glass pipette prior to transferring the embryos to prevent them from sticking to the glass pipette.

Peeling younger embryos (<20h AEL at 27°C)

63

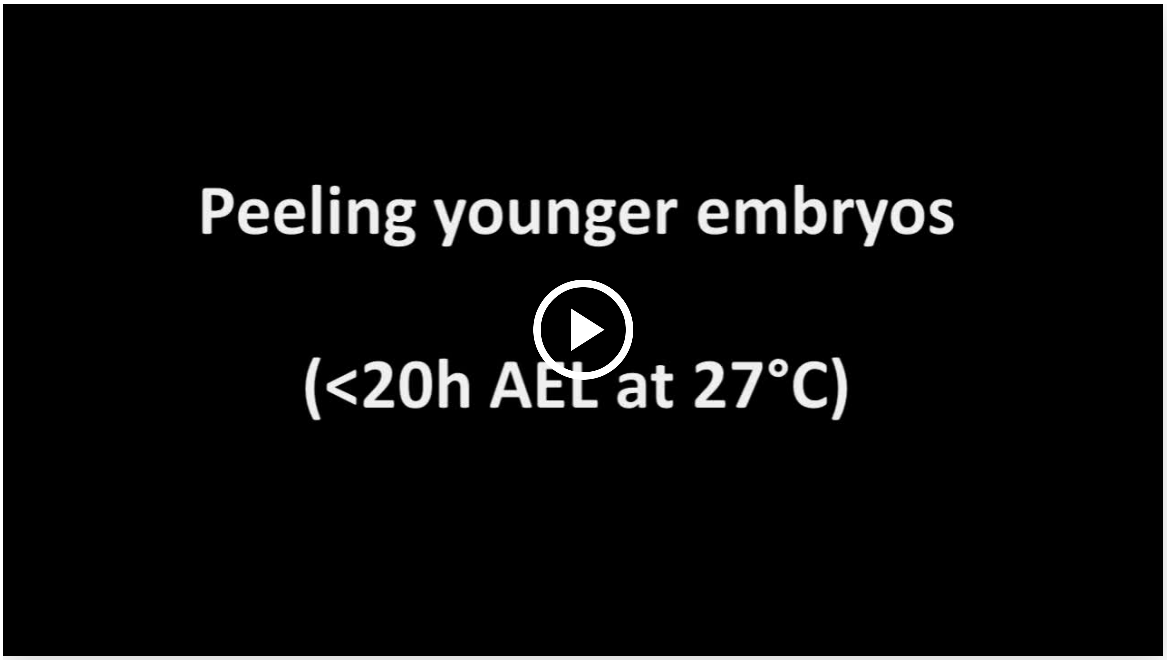

Note

This protocol has been successful for peeling embryos as young as 14h AEL at 27°C.

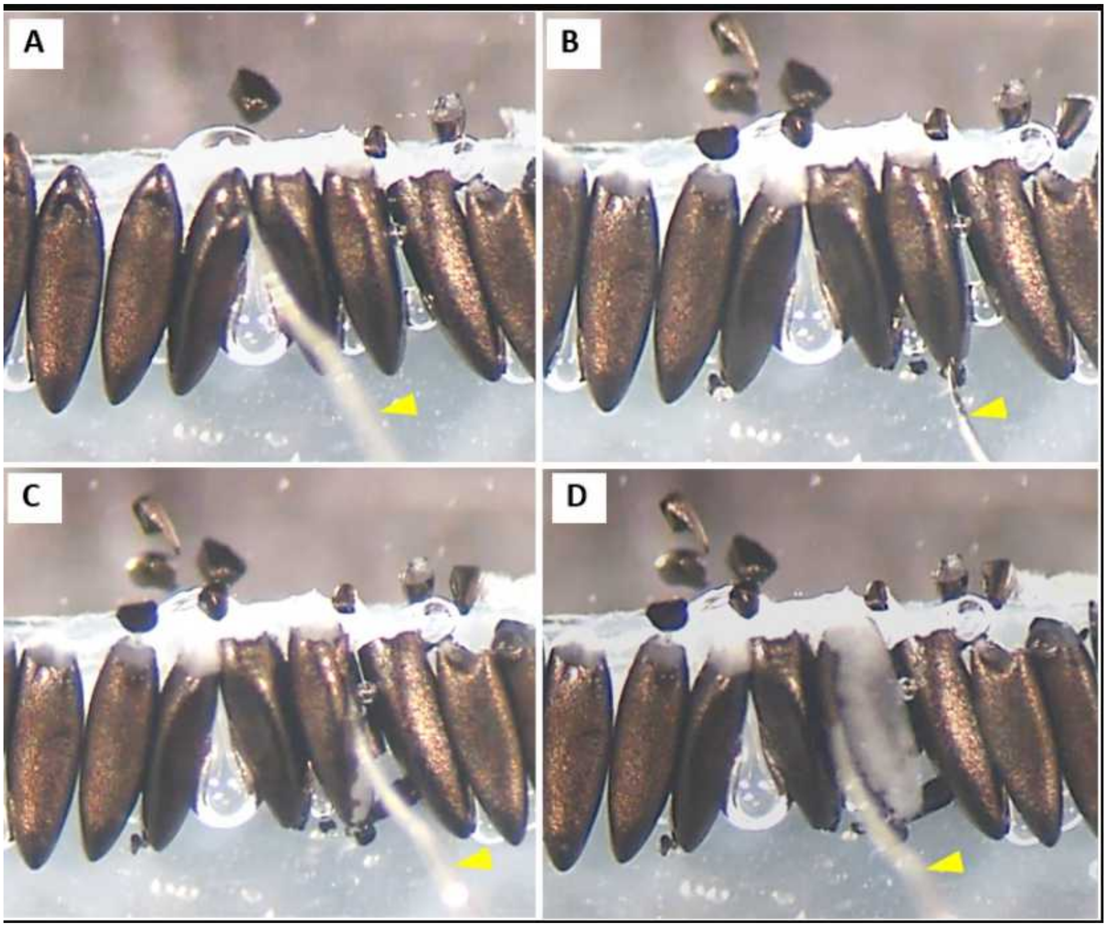

Figure 10. Intermediate and final steps in the removal of the vitelline membrane for younger embryos following removal of the anterior end "cap" of the embryo. (A) Removal of the anterior end with the tungsten wire probe; (B) Cutting of the posterior end of the embryo; (C) Dissection of the vitelline membrane along the sagittal plane; (D) Release of the embryo by folding back the vitelline membrane. The yellow arrows indicate the tungsten wire probe. Follow steps 39 to 57 for the initial steps of peeling younger embryos.

Note

Younger embryos can be peeled identically to older embryos with the exception of being extruded using the Roboz tool.

Tear a small opening in the posterior of the embryo using the tungsten wire probe.

64 Insert the tungsten wire probe into the posterior opening of the vitelline membrane and carefully tear upwards in a stepwise fashion until the entire vitelline membrane has been dissected along the sagittal plane.

Note

- Do this step incrementally to prevent tearing the embryo.
- The stab knife can also be used to dissect the length of the vitelline membrane.

65 Press the edges of the vitelline membrane into the tape adhesive to free the embryo.

Note

Do this step incrementally, alternating between left and right sides of the embryo if needed.

66 Collect embryos into a pile and transfer into a 1.5 mL tube using a Pasteur pipette.

Note

Wet the glass pipette prior to transferring the embryos to prevent them from sticking to the glass pipette.

67 Store all peeled embryos at 4 °C and process for staining within one day for best results.

Protocol references

1. Clemons A, Haugen M, Flannery E,Kast K, Jacowski C, Severson D, Duman-Scheel M. 2010. Fixation and Preparation of Developing Tissues from Aedes aegypti. Cold Spring Harb Protoc. doi:10.1101/pdb.prot5508.
2. Schember I, Reid W, Halfon MS. 2023. Conserved and novel enhancers regulate expression of Aedes aegypti single-minded in the embryonic ventral midline. bioRxiv 2023.08.01.551414; doi: https://doi.org/10.1101/2023.08.01.551414.
